# Six Heterocyclic Metabolites from the Myxobacterium *Labilithrix luteola*

**DOI:** 10.3390/molecules23030542

**Published:** 2018-02-28

**Authors:** Lucky S. Mulwa, Rolf Jansen, Dimas F. Praditya, Kathrin I. Mohr, Joachim Wink, Eike Steinmann, Marc Stadler

**Affiliations:** 1 Department of Microbial Drugs, Helmholtz Centre for Infection Research and German Centre for Infection Research (DZIF), partner site Hannover/Braunschweig, Inhoffenstrasse 7, 38124 Braunschweig, Germany; luckymulwa@gmail.com (L.S.M.); Rolf.Jansen@helmholtz-hzi.de (R.J.); |Kathrin.Mohr@helmholtz-hzi.de (K.I.M.); 2 Work group Microbial Strain Collection (MISG), Helmholtz Centre for Infection Research, Inhoffenstrasse 7, 38124 Braunschweig, Germany; Joachim.Wink@helmholtz-hzi.de; 3 TWINCORE—Centre for Experimental and Clinical Infection Research (Institute of Experimental Virology) Hanover. Feodor-Lynen-Str. 7–9, 30625 Hannover, Germany; dimas.praditya@twincore.de (D.F.P); eike.steinmann@twincore.de (E.S.)

**Keywords:** antiviral activity, antimicrobial activity, fermentation, HCV, natural products, nitroindole

## Abstract

Two new secondary metabolites, labindole A [2-methyl-3-(2-nitroethyl)-3H-indole] (**1**) and labindole B [2-methyl-3-(2-nitrovinyl)-3H-indole] (**2**), were isolated from the myxobacterium *Labilithrix* *luteola* (DSM 27648^T^). Additionally, four metabolites **3**, **4**, **5** and **6** already known from other sources were obtained. Their structures were elucidated from high resolution electrospray ionisation mass spectrometry (HRESIMS) and 1D and 2D nuclear magnetic resonance (NMR) spectroscopy data and their relative configuration was assigned based on nuclear Overhauser effect (NOE) and vicinal ^1^H-NMR coupling data. The compounds where tested for biological activities; labindoles A (**1**) and B (**2**) exhibited significant activity against Hepatitis C Virus, 9*H*-carbazole (**3**), 3-chloro-9*H*-carbazole (**4**) and 4-hydroxymethyl-quinoline (**5**) showed antifungal activities. Moreover, compound **3** had weak to moderate antibacterial activities, while labindoles A (**1**) and B (**2**) were devoid of significant antifungal and antibacterial effects.

## 1. Introduction

The global challenge of increased drug resistance has led to strong demand to increase the chemical diversity of antibiotics, especially to obtain drugs that can overcome bacterial resistance through new modes of action [1]. The emergence of old viral infections such as Chikungunya, an RNA virus that belongs to the alphavirus genus of the family Togaviridae, a mosquito-borne viral disease first described during an outbreak in southern Tanzania in 1952, as well as Ebola outbreaks [2] and HIV pandemics, call for more research into antiviral drugs. Myxobacteria have emerged to be among the main producers of bioactive molecules, with a high chemical diversity in their unique structures and unusual and often novel modes of action [3]. In recent years, various novel carbon skeletons with interesting bioactivities have been isolated from hitherto under-explored taxa such as sorazolones [4] and carolacton [5] from *Sorangium cellulosum*, argyrins [6] and tubulysins [7] from *Archangium gephyra*, aetheramides from *Aetherobacter* spp. [8], disciformycins from *Pyxidicoccus fallax* [9] and cystobactamids from *Cystobacter* spp. [10]. This indicates that isolation of compounds from uncommon strains, in particular those that belong to unexplored phylogenetic lineages, such as *Labilithrix*, is of great interest. During the course of our studies on the secondary metabolism of the recently described myxobacterium *Labilithrix luteola* (DSM 27648^T^) [11], which belongs to the family Labilitrichaceae in suborder Sorangiineae, antimicrobial activities were observed and correlated with some prominent peaks with unique high performance liquid chromatography-diode array/mass spectrometry (HPLC-DAD/MS) characteristics attracted our interest. In the following, the fermentation process was optimized and scaled up, resulting in the discovery of two new and several known compounds. Their isolation and biological and physico-chemical characterization are reported in the current paper.

## 2. Results and Discussion

In batches of 500 mL broth in 1 L shake flasks, ten liters of fermentation broth of *Labilithrix luteola* was incubated at 30 °C in presence of Amberlite XAD 16 (Rohm and Haas, Frankfurt, Germany) absorber resin [12] and harvested after 14 days. The resin was recovered by filtration and eluted with methanol, giving 5 g of crude extract, which was separated by Sephadex LH-20 gel permeation chromatography in methanol. Subsequent preparative reversed-phase HPLC of the LH-20 fractions gave six hetero-aromatic metabolites.

**Figure 1:**
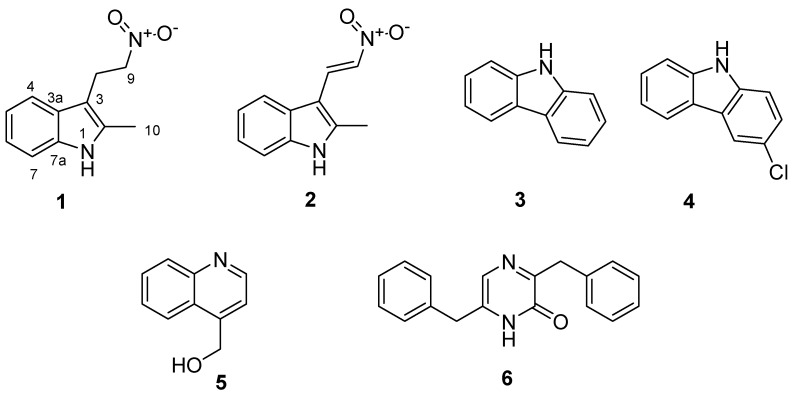
Secondary metabolites isolated from *Labilithrix luteola*.

Compound **1** (Figure 1) was obtained as yellow powder and turned bright orange on spraying thin layer chromatography plates with Dragendorff reagent [13]. The ultraviolet (UV) absorption maxima at 222 nm and 281 nm suggested the presence of an indole alkaloid. The elemental formula was determined by high resolution electrospray ionisation mass spectrometry (HRESIMS) combined with isotopic pattern analysis of the molecular ion cluster [M + H]^+^ (*m*/*z* 205.0968) as C_11_H_12_N_2_O_2_, revealing seven double-bond equivalents (DBE). ^13^C and ^13^C-DEPT (Distortionless Enhancement by Polarization Transfer) NMR spectra in CD_3_OD indicated four quaternary carbons as well as four aromatic methine, two methylene, and one methyl carbon (Table 1). The carbons were correlated to their corresponding 11 protons from an ^1^H, ^13^C-HSQC spectrum, which left one exchangeable proton. The ^1^H, ^1^H-COSY-NMR spectrum showed that the four aromatic methines belong to a ortho disubstituted aromatic ring, and the methylene groups were directly connected, while the methyl group only had a long-range correlation with CH_2_-8. These structure elements were connected with the remaining carbons due to their ^1^H, ^13^C-HMBC correlations to give the carbon skeleton of **1**. The ^1^H, ^15^N-HMBC spectrum revealed a nitrogen signal at δ_N_ = 133.1 correlated to methyl group C-2 and methine C-7 completing an indole group, while the second nitrogen was attached as a nitro group, δ_N_ = 388.24 ppm, to methylene C-9 from correlations with C-8 and C-9. Although the chemical shift δ_N_ = 388.24 ppm was in the expected range, the presence of the nitro group was further verified by a very strong band at 1556 cm^−1^ in the IR spectrum and by a fragment ion [M+H-HNO_2_]^+^ in the HR-ESIMS at *m*/*z* 158.0962. Finally, the exchanged NH proton completed the structure of Labindole A (**1**) as 2-methyl-3-(2-nitroethyl)-1*H*-indole.

Compound **2** was isolated by reverse phase HPLC from another LH-20 chromatography fraction of the raw extract as yellow powder and tested positive to Dragendorff reagent. Analysis of the monoisotopic ion clusters [M + H]^−^ and [M + Na + H]^−^ (203.0814 and 225.0634) suggested the elemental formula C_11_H_10_N_2_O_2_, i.e., a loss of two hydrogens compared to **1**. The dehydrogenation was also apparent from the NMR spectra of **2**, which had lost the methylene groups, but showed two new methines at δ_H/C_ = 8.35/132.6 ppm and 7.80/131.8 ppm (Table 1). Their vicinal coupling constant of *J* = 13.3 Hz indicated a trans configuration of the new Δ^8,9^ double bond in Labindole B (**2**), i.e., (*E*)-2-methyl-3-(2-nitrovinyl)-1*H*-indole. 

The extracts of *Labilithrix luteola* (DSM 27648) further contained four hetero-aromatic metabolites previously isolated from other sources, including 9*H*-carbazole (**3**) [14], which was previously isolated from root bark of *Glycomis pentaphylla* [15], while 3-chloro-9*H*-carbazole (**4**) was found in bovine urine [16]. Interestingly, 4-hydroxymethyl-quinoline (**5**) was already known from the Myxobacterium *Archangium gephyra* [17] and from the basidiomycete fungi “*Polyporus”* (current generic name: *Pycnoporus*) *sanguineus* and “*Polyporus”* (current generic name: *Coriolus*) *versicolor* [18] with moderate activity against yeast and filamentous fungi, whereas 3,6-dibenzylpyrazin-2(1*H*)-one (**6**) was recently described as a product of the expression of gene clusters from gut bacteria [19]. 

**Figure 2:**
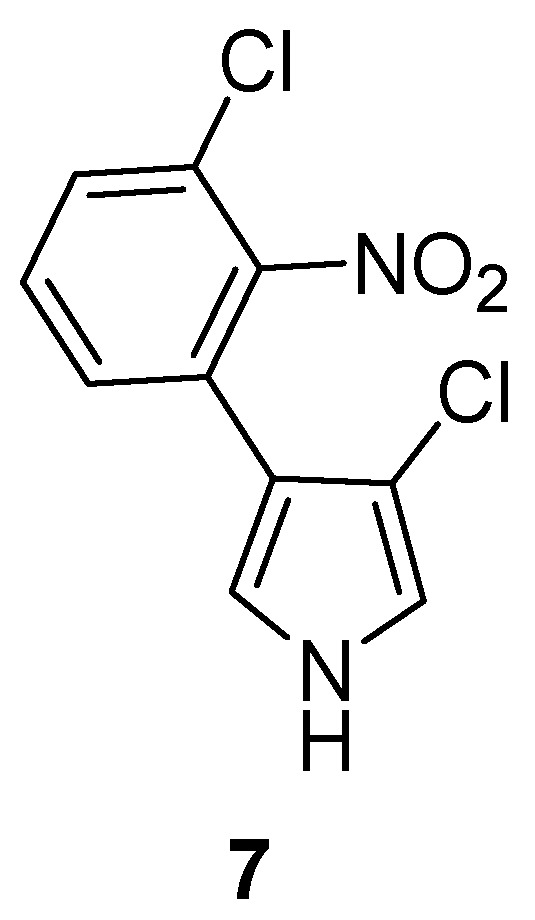
Chemical structure of pyrrolnitrin (**7**).

Although previously reported as chemically derivative compounds, this is the first isolation of the structurally related Labindoles A (**1**) [20] and B (**2**) [21] from a natural source. Interestingly, the 2-methylindole residue had previously only been found in 2-methyltryptophan as an intermediate in the biosynthesis of the peptide antibiotic thiostrepton in trapping experiments with *Streptomyces laurentii* [22]. However, an aromatic nitro compound, pyrrolnitrin (**7**) (Figure 2) has previously been described as a fungistatic antibiotic of the myxobacteria *Myxococcus fulvus*, *Corallococcus exiguus*, and *Cystobacter ferrugineus* [23], and it is also known to be an antifungal agent of pseudomonads [24]. Labindoles A (**1**) and B (**2**) are very rare examples of primary nitro compounds from bacteria. 

**Figure 3:**
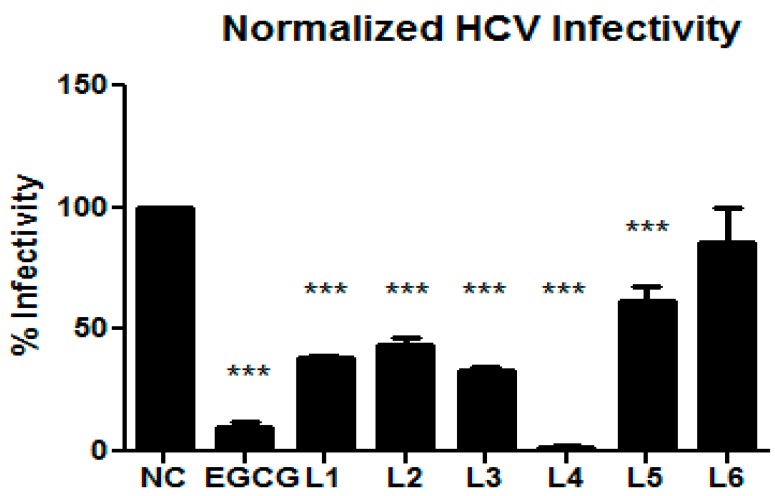
Hepatitis C Virus (HCV) assay: NC-Negative control, EGCG-Positive control, L1-Labindole A (**1**), L2-Labindole B (**2**), L3–L6 compound **3**–**6**. The assay was performed in quadruplicate (L1–L2) and triplicate (L3–L6) and is presented as the mean ± standard deviation. *** *P* ≤ 0.05. Huh-7.5. Cells were inoculated with RLuc Jc1 reporter viruses in the presence of different compounds. The inoculum was removed 4 h later and monolayers were washed three times with phosphate buffered saline (PBS) and overlaid with fresh medium containing no inhibitors. Infected cells were lysed 3 days later, and reporter virus infection was determined by renilla luciferase activity. The cell viability was measured by determination of firefly luciferase. Viability assay results are given in supplementary material (Figure S19).

The compounds isolated were tested in our screening panel against bacteria, fungi, cell cultures and for antiviral activity against HCV (Hepatitis-C-Virus) in human liver cells. Compound **4** inhibited HCV infectivity very strongly. Compound **3**, Labindoles A (**1**) and B (**2**) had a statistically significant HCV infectivity inhibition, **5** had a weak activity, while **6** was not active, all showed no cytotoxicity on the liver cells, which was simultaneously determined (Figure 3). 

The green tea molecule Epigallocatechin gallate (EGCG) was used as positive control [25]. Although **4** has been previously isolated^16^, this is the first time the strong inhibition of HCV infectivity activity has been reported. 

When **1**–**6** were tested for their cytotoxicity against growing primary and cancer cells lines, they did not show any significant activity (Table 2). All compounds were analyzed for their antimicrobial activity against bacteria and fungi. Labindole A (**1**), Labindole B (**2**) and **6** showed no activity, whereas **3**, **4**, and **5** showed weak antifungal activity in addition **3** had weak activity against *Bacillus subtilis* and *Chromobacter violaceum* (Table 3).

## 3. Materials and Methods 

### 3.1. General Experimental Procedures

Analytical TLC: aluminum sheets silica gel Si 60 F254 (Merck, Darmstadt, Germany), detection by UV absorption at 254 nm and 366 nm, Dragendorff spray reagent (alkaloids) [13]. UV data were recorded on a Shimadzu UV/vis-2450 spectrophotometer using methanol (UVASOL, Merck, Darmstadt, Germany) as solvent. NMR spectra were recorded on Bruker Avance DMX 600 or Ascend DPX 500/700 (Bruker Biospin, Bremen, Germany) NMR spectrometers, locked to the deuterium signal of the solvent. Data acquisition, processing, and spectral analysis were performed with standard Bruker software and ACD/NMR workbook. Chemical shifts are given in parts per million (ppm), and coupling constants in hertz (Hz). HRESIMS data were recorded on a maXis ESI QTOF mass spectrometer (Bruker Daltonics, Bremen, Germany); molecular formulas were calculated using the isotopic pattern (Smart Formula algorithm). Analytical RP-HPLC was carried out with an Agilent 1260 system equipped with a diode-array UV detector and a Corona Ultra detector (Dionex, Germering, Germany). HPLC conditions: column 125 × 2 mm, Nucleodur C_18_, 5 μm (Macherey-Nagel, Düren, Germany), solvent A: 5% MeCN in water, 5 mmol NH_4_Ac, 0.04 mL/L AcOH; solvent B: 95% MeCN, 5 mmol NH_4_Ac, 0.04 mL/L AcOH; gradient system: 10% B increasing to 100% B in 30 min, 100% B for 10 min, to 10% B post-run for 10 min; 40 °C; flow rate 0.3 mL/min.

### 3.2. Cultivation of Labirithrix luteola

The type strain of *Labirithrix luteola* (designated B00002^T^) was isolated from forest soil samples collected from Yakushima Island, Kagoshima, Japan [11]. A sample was deposited at DSMZ (Braunschweig, Germany), collection number DSM 27648 (GenBank accession number of 16S rRNA gene: AB847449). It was reactivated in 20 mL of liquid medium composed of 0.1% soya meal, 0.15% Casiton, 0.1% yeast extract, 0.1% CaCl_2_, 50 mM HEPES (11.9 g), 0.1% glucose, 0.4% starch, 0.5% MgSO_4_ and 4 mg/L Fe-EDTA (ethylene diamine tetraacetate), and maintained at pH 7.4. The culture was scaled up to 1 L and used to inoculate 10 L media in 1 L shake flasks with 500 mL of medium and 2% Amberlite XAD 16 adsorber resin as described previously [12]. The medium was composed of 0.4% de-fatted soya meal flour, 0.4% skim milk powder, 1% starch, 50 mM HEPES (11.9 g), 0.1% MgSO_4_, 0.2% yeast extract, 8 mg Fe-EDTA, 0.5% glycerin and maintained at pH 7.4. Each flask was inoculated with 1 mL of well-grown 5-day culture and then incubated at 30 °C for 14 days. 

### 3.4. Isolation of Secondary Metabolites

The XAD resin was recovered by sieving and extracted in a glass column (70 × 8 cm) with methanol (approximately 3 L) followed by acetone (1 L) at a flow rate of 30 mL/min. The solvents were evaporated in a rotatory evaporator, giving 5 g of crude extract. The crude extract divided into five portions and separated using Sephadex LH 20 in methanol, flow rate 8 mL/min.; detection UV at 280 nm (Pharmacia Biotec, Piscataway, NJ, USA). The fractions were combined peak-wise, giving 18 fractions, which were evaporated to dryness. 

Fraction 14 (15 mg) was further purified by preparative RP-HPLC (Nucleodur, C18, 5 µm column, 250 × 21 mm, water and MeCN solvent system at gradient of 30–100% MeCN in 30 min.; flow rate 20 mL/min, UV detection at 300 nm. Labindole A (**1**) (10 mg) eluted with a retention time of *t_R_* = 29.9 min. 

Fraction 17 (41.6 mg) was separated by RP-HPLC (solvent system MeCN/Water gradient 45–100% MeCN in 30 min) to give labindole B (**2**) (22.8 mg, *t_R_* = 22.3 min) and 9*H*-carbazole (**3**) (10.3 mg, *t_R_* = 13.9 min). 

Fraction 18 (13.6 mg) yielded 4 (6.8 mg, *t_R_* = 31.1 min.) upon separation by RP-HPLC using a Methanol/Methanol:Water (1:1) solvent system (70–100% Methanol).

Fraction 11 (151 mg) was dissolved in about 10 mL of methanol and centrifuged for 5 min at 3000 rpm. The supernatant was concentrated to 3 mL and separated by RP-HPLC (Nucleodur C18 column, 250 × 20 mm, solvent system MeCN /water, gradient 30–100% MeCN in 1 h; flow rate 20 mL/min; UV detection at 220 nm, 254 nm and 300 nm) yielding 5 (12.7 mg, *t_R_* = 6.2 min) and 6 (11.9 mg, *t_R_* = 25.0 min). The sediments were decanted and washed with methanol and identified by NMR as 6 (8.8 mg) giving a total of 20.7 mg.

*Labindole A* (**1**): C_11_H_12_N_2_O_2_ M = 204.22; UV (Methanol) λ_max_ (log ε): 281 (3.391), 222 (4.049), 208 (3.940) nm with a shoulder at 291 (3.318) nm; IR (KBr): ν = 1556 (s), 753 (s) cm^−1^; NMR data in Table 1 and Table S1. HR-ESIMS: [M + H]^+^ *m*/*z* 205.0968, calcd. 205.0971, [M + H − HNO_2_]^+^ *m*/*z* 158.0962, calcd. 158.0964.

*Labindole B* (**2**): C_11_H_10_N_2_O_2_ M = 202.21; UV (Methanol) λ_max_ (log ε): 335 (2.398), 322 (2.463), 292 (3.111), 256 (3.159), 233 (3.516), 210 (3.436), 331 (2.343), 305 (2.343), 267 (2.544), 251 (3.065), 217 (3.400) nm; IR (KBr): ν = 1451 (s), 1328 (s), 749 (s), 726 (s) cm^−1^; NMR data in Tables 1 and S2. HR-ESIMS: [M + H]^+^ *m*/*z* 203.0814, calcd. 203.0815, [M + Na]^+^ *m*/*z* 225.0634, calcd. 225.0634.

### 3.5. Inhibitory Effects on HCV Infectivity

Huh7.5 cells stably expressing Firefly luciferase (Huh7.5 Fluc) were cultured in Dulbecco’s modified minimum essential medium (DMEM, Life Technologies, Carlsbad, CA, USA) containing 2 mM glutamine, 1× minimum essential medium nonessential amino acids (MEM NEAA, Life Technologies), 100 μg/mL streptomycin, 100 IU/mL penicillin (Life Technologies), 5 μg/mL blasticidin and 10% fetal bovine serum. Cells were maintained in a 37 °C environment with 5% CO_2_ supply. Cells were infected with Jc1-derived Renilla reporter viruses in the presence or absence of compounds as described previously [25]. Infected cells were lysed and then frozen at −80 °C for 1 h following measurements of Renilla and Firefly luciferase activities on a Centro XS3 Microplate luminometer (Berthold Technologies, Bad Wildbad, Germany) as indicators of viral genome replication and cell viability, respectively.

### 3.6. Cytotoxic Activity

The cytotoxicity of the test compounds was evaluated by measuring the effect produced on cell morphology, including the nuclei and cell growth in vitro. Cell monolayers were prepared in 24-well tissue culture plates and exposed to various concentrations of the compounds. Plates were checked by light microscopy after 24, 48 and 72 h. Cytotoxicity was scored as morphological alterations (e.g. rounding up, shrinking, detachment and disintegration of nuclei). The viability of the cells was determined by a tetrazolium-based colorimetric method using 3-(4,5-dimethylthiazol-2-yl)-2,5-diphenyltetrazolium bromide (MTT), as previously described [26,27]. The 50% cytotoxic dose, CD_50_, is the concentration of the compound that reduced the absorbance of the control sample by 50%

### 3.7. Antimicrobial Testing

Aliquots of 2 and 20 µL (conc. 1 mg/mL) of compounds **1**–**6** and reference drugs were tested against three fungi, three Gram-positive bacteria and three Gram-negative bacteria (Table 3). The minimum inhibitory concentration (MIC) values were determined in 96-well microtiter plates by 1:1 serial dilution in EBS medium (0.5% casein peptone, 0.5% protease peptone,0.1% meet extract, 0.1% yeast extract, pH 7.0) for bacteria and MYC medium (1.0% glucose, 1.0% phytone peptones, 50 mM HEPES (11.9g/L), pH 7.0) for fungi, as previously described [28]. The lowest concentration of the drug preventing visible growth of the pathogen was taken as the MIC.

## 4. Conclusions

Two new secondary metabolites, labindoles A and B, were isolated from *Labilithrix luteola* (DSM 27648^T^) in addition to four known compounds. Their structure elucidation was achieved by combination of spectroscopic methods including MS, NMR, UV and IR. Compound **4** inhibited HCV infectivity very strongly. Compound **3**, Labindoles A (**1**) and B (**2**) had a statistically significant HCV infectivity inhibition, **5** had a weak activity, while **6** was not active. Antiviral activities against other viruses will be investigated in the future.

## Figures and Tables

**Table 1:** ^1^H, ^13^C, and ^15^N-NMR spectral data of Labindoles A (**1**) and B (**2**).

Position	1 ^a^		2 ^b^	
	δ_C(N)_	δ_H_ m (*J* [Hz])	δ_C(N)_	δ_H_ m (*J* [Hz])
1	(133.1)		(141.4)	8.56 br. s
2	134.3		144.2	
3	106.0		106.5	
3a	129.4		125.7	
4	118.1	7.41 *dt* (7.7, 0.95)	120.1	7.71 *m*
5	120.0	6.97 *ddd* (7.8, 7.0, 1.4)	122.6	7.30 *m*
6	121.8	7.02 *ddd* (8.1, 7.0, 1.2)	123.6	7.29 *m*
7	111.6	7.23 *dt* (7.9, 0.9)	111.4	7.38 *m*
7a	137.3		135.9	
8	24.0	3.38 *t* (7.1)	132.6	8.35 *d* (13.3)
9	76.8	4.62 *t* (7.3)	131.8	7.80 *d* (13.3)
10	11.3	2.35 *s*	12.5	2.66 *s*
11	(388.2)		(375.3)	

^a 1^H/^13^C/^15^N at 500/125.8/50.7 MHz in CD_3_OD; ^b 1^H/^13^C/^15^N-NMR at 500.3/125.8/50.7 MHz in CDCl_3_.

**Table 2:** Cytotoxicity (µM) of compounds **1**–**6**.

Cell Line	1	2	3	4	5	6
Mouse fibroblasts (L929) ^a^	-	-	45	44	-	-
Human nasopharyngeal cells (KB3.1)^a^	-	-	60	42	16	-

Superscript represents control: **^a^** Epothilone B.

**Table 3:** Antimicrobial activity (µg/mL) of compounds **1**–**6.** Methanol was used as a negative control.

Test Strain	1	2	3	4	5	6	Methanol
**Fungi**							
*Mucor hiemalis* (DSM 2656) ^b^	-	-	16.6	33.3	33.3	-	-
*Candida albicans* (DSM1665) ^b^	-	-	33.3	33.3	33.3	-	-
*Pichia anomala* (DSM6766) ^b^	-	-	67	16.6	-	-	-
**Gram positive bacteria**							
*Staphylococcus aureus* (Newman) ^c^	-	-	-	-	-	-	-
*Bacillus subtilis* (DSM 10) ^d^	-	-	6.7	-	-	-	-
*Micrococcus luteus* (DSM 1790) ^e^	-	-	-	-	-	-	-
**Gram negative bacteria**							
*Escherichia coli* (ToLC) ^c^	-	-	33.3	-	-	-	-
*Escherichia coli* (DSM1116) ^c^	-	-	-	-	-	-	-
*Chromobacter violaceum* (DSM 30191) ^d^	-	-	6.7	-	-	-	-

Superscripts represent positive controls as follows: ^b^ Nystatin, ^c^ Gentamycin, ^d^ Tetracycline, ^e^ Ampicillin.

## Supplementary Materials

The following are available online, Tables of NMR data and figures of the ^1^H and ^13^C-NMR spectra of **3**, **4**, **5** and **6** and the HCV infectivity results showing viability are available in the supplemental material.
