# Supplementary material for: Six Heterocyclic Metabolites from the Myxobacterium Labilithrix luteola
Source: Molecules. 2018 Feb 28;23(3):542. doi: 10.3390/molecules23030542 (PMC6017429; doi:10.3390/molecules23030542)
Supplement: Supplementary file 1 [file molecules-23-00542-s001.pdf]

Supplemental Information

Six Heterocyclic Metabolites from the Myxobacterium *Labilithrix luteola*

Lucky S. Mulwa[1,2], Rolf Jansen[1], Dimas F. Praditya[3], Kathrin I. Mohr[1,2], Joachim Wink[2], Eike Steinmann[3] and Marc Stadler [1,*]

[1] Department of Microbial Drugs, Helmholtz Centre for Infection Research and German Centre for Infection

Research (DZIF), partner site Hannover/Braunschweig, Inhoffenstrasse 7, 38124 Braunschweig, Germany; luckymulwa@gmail.com (L.S.M.); Rolf.Jansen@helmholtz-hzi.de (R.J.); dimas.praditya@twincore.de (D.F.P); Kathrin.Mohr@helmholtz-hzi.de (K.I.M.); eike.steinmann@twincore.de (E.S.); Joachim.Wink@helmholtz-hzi.de (J.W); Marc.Stadler@helmholtz-hzi.de (M.S.)

[2] Work group Microbial Strain Collection (MISG), Helmholtz Centre for Infection Research, Inhoffenstrasse 7, 38124 Braunschweig, Germany

[3] TWINCORE- Centre for Experimental and Clinical Infection Research (Institute of Experimental Virology) Hanover. Feodor-Lynen-Str. 7-9, 30625 Hannover, Germany. Tel: +49(0)511-220027-133

[*] Correspondence: marc.stadler@helmholtz-hzi.de; Tel.: +49 531 6181-4240; Fax: +49 531 6181 9499

Table S1. NMR data of Labindole A (**1**) in CD_3OD.

Pos.	$\delta_{C(N)}$	XHn	δ_H	mult. (J [Hz])	COSY	N/ROESY	H in HMBC
1	(133.1)	NH					7, 10
2	134.3	C					10, 8
3	106.03	C					10, 8, 9, 4
3a	129.4	C					10, 8, 5, 7, 4
4	118.1	CH	7.41	dt (7.7, 0.95)	5, 7	8, 5, 9	6
5	120.0	CH	6.97	ddd (7.8, 7.0, 1.4)	6, 4	4	7
6	121.8	CH	7.02	ddd (8.1, 7.0, 1.2)	5, 7	7	5, 4
7	111.6	CH	7.23	dt (7.9, 0.93)	6, 4	6	5, 6, 4
7a	137.3	C					6, 4
8	24.0	CH_2	3.38	t (7.10)	10, 9	10, 9, 4	9
9	76.8	CH_2	4.62	t (7.32)	8	8, 4	8
10	11.3	CH_3	2.35	s	8	8	-
11	(388.2)	N					9, 8

$^1H/^{13}C/^{15}N$ at 500/125.8/50.7 MHz

Figure S1. NMR correlations Labindole A (**1**)

(blue COSY, green HMBC, purple N/ROESY)

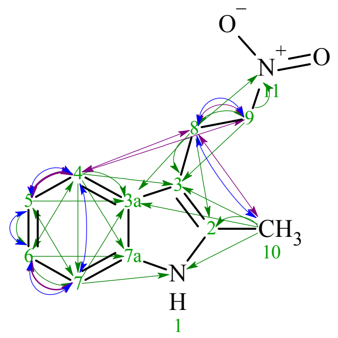

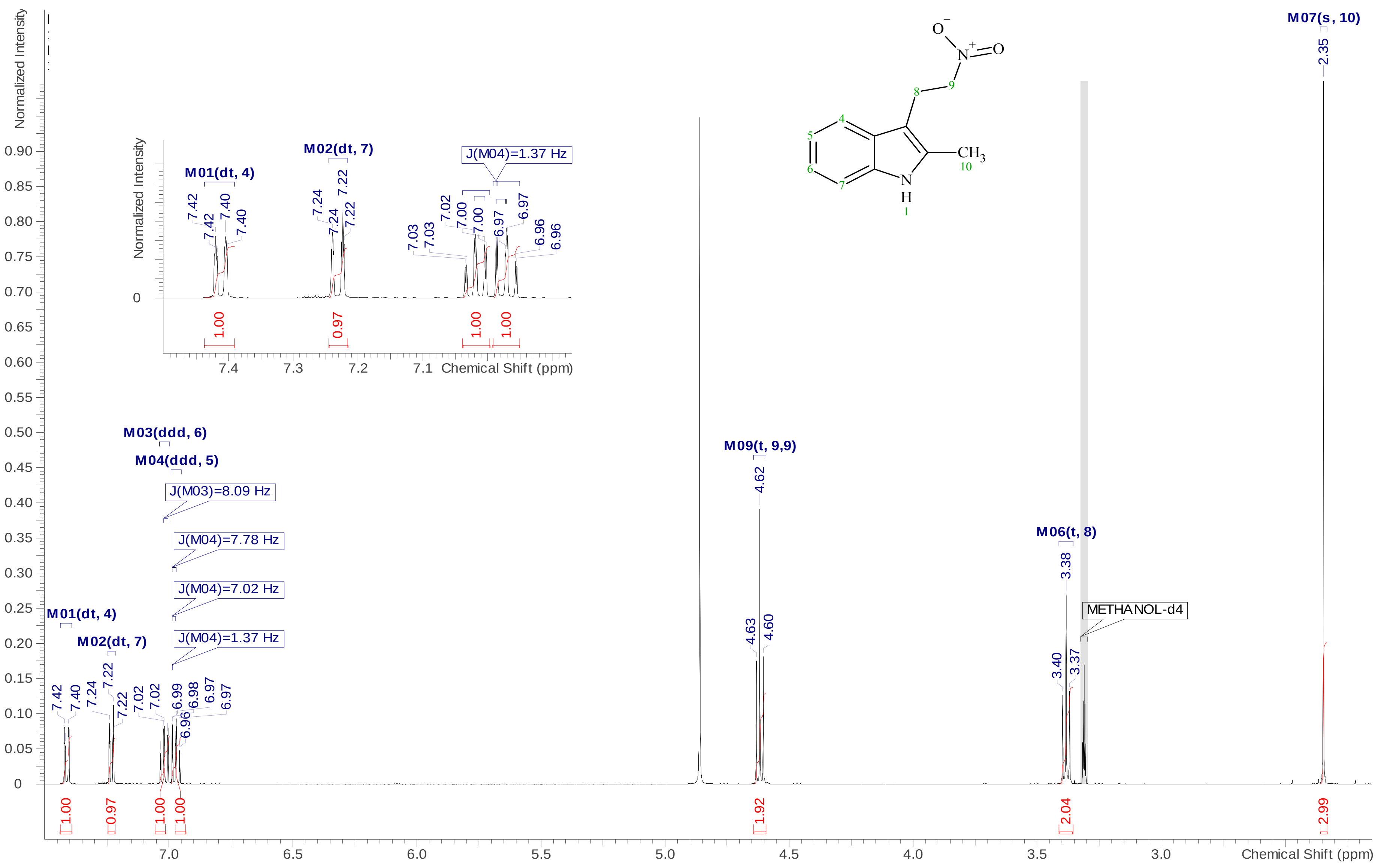

Figure S2. ^{1}H NMR spectrum of 2-methyl-3-(2-nitro-ethyl)-1H-indole (**1**) in CD_3OD

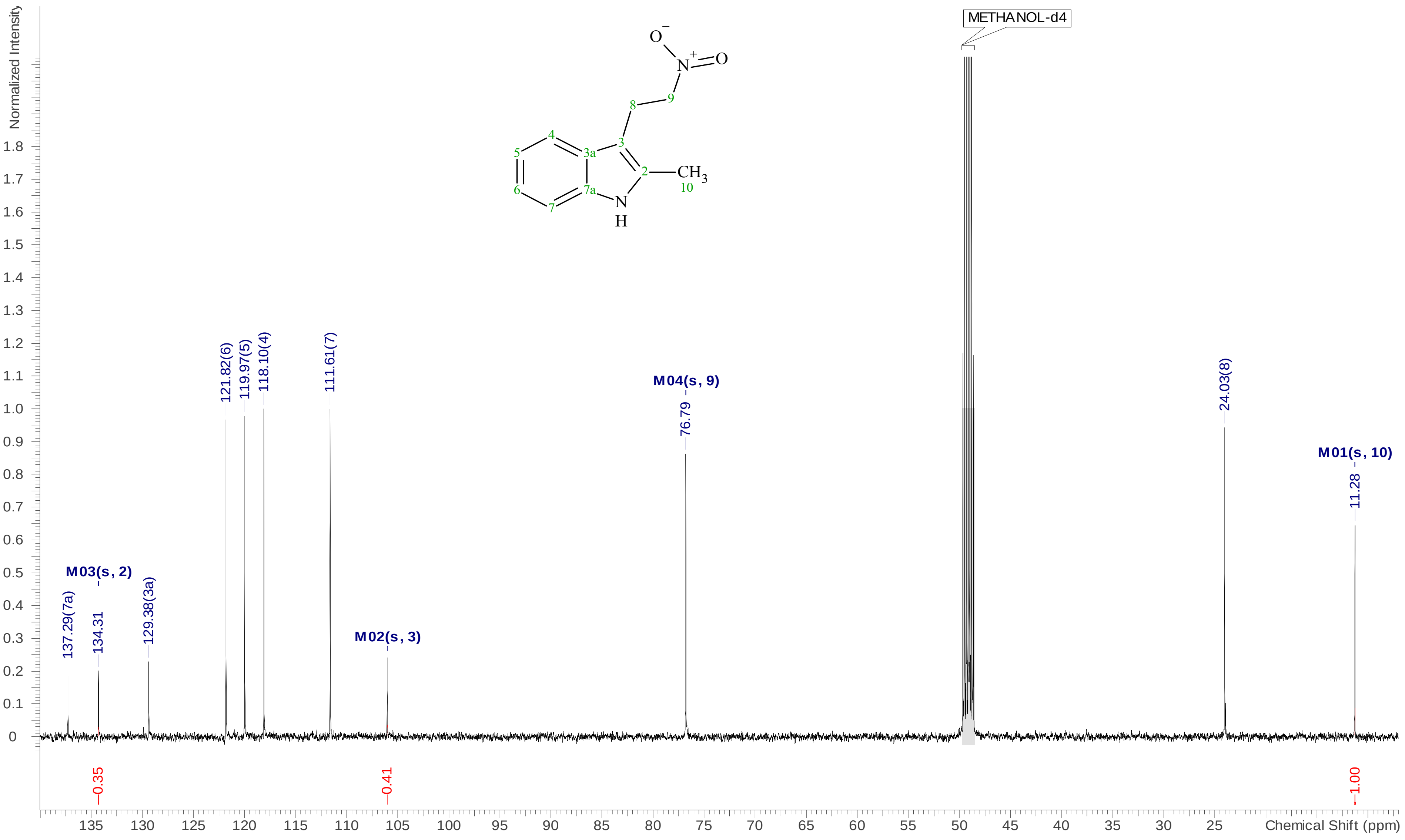

Figure S3. ^{13}C NMR spectrum of 2-methyl-3-(2-nitro-ethyl)-1H-indole (**1**) in CD_3OD.

Table S2. NMR data of Labindole B (**2**) in $CDCl_3$.

Pos.	$\delta_{C(N)}$	XHn	δ_H	mult. (*J* [Hz])	COSY	N/ROESY	H in HMBC
1	(141.4)	NH	8.56	br s		10, 7	7, 10
2	144.2	C					10, 8
3	106.5	C					10, 4, 9
3a	125.7	C					10, 5, 7, 4, 8
4	120.1	CH	7.71	m	5, 7	5, 9	6
5	122.6	CH	7.30	m	6, 4	4	7
6	123.6	CH	7.29	m	5, 7		4
7	111.4	CH	7.38	m	6, 4	1	5
7a	135.9	C					6, 4
8	132.6	CH	8.35	d (13.3)	9	10	9
9	131.8	CH	7.80	d (13.3)	8	4, 10	8
10	12.5	CH_3	2.66	s	8	8, 9, 1	
11	(375.3)	N					9, 8

$^1H/^{13}C/^{15}N$ NMR at 500.3/125.8/50.7 MHz

Figure S4. Correlations in the NMR data of Labindole B (**2**)

(blue and red COSY, green HMBC)

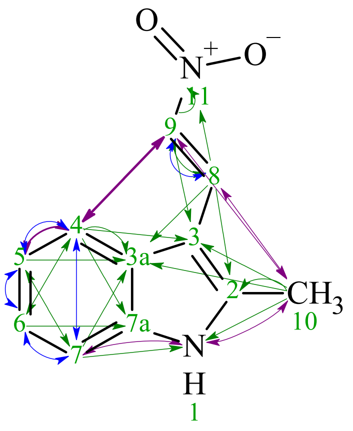

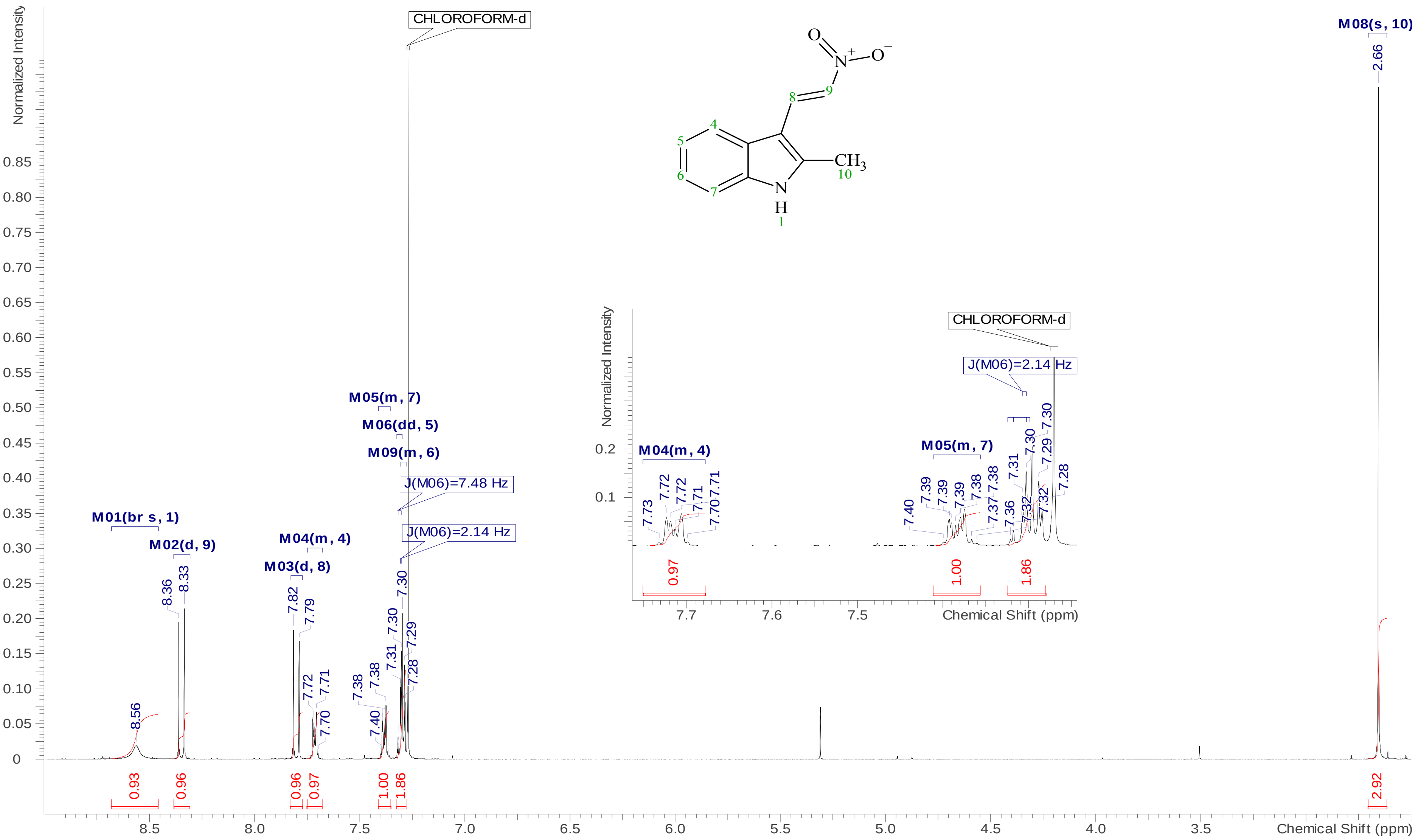

Figure S5. ^{1}H NMR spectrum of 2-methyl-3-(2-nitro-vinyl)-1*H*-indole (**2**) in $CDCl_3$.

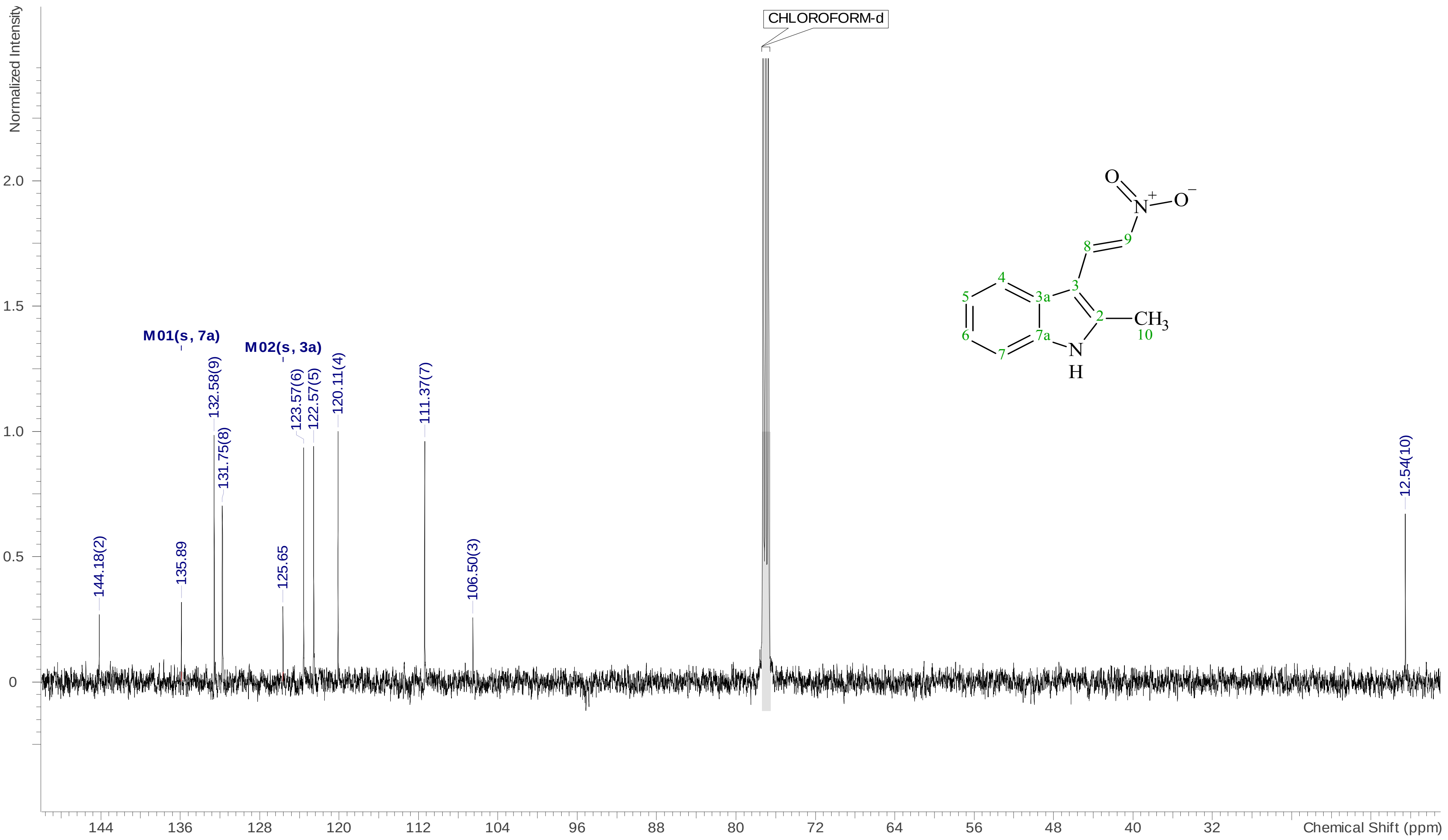

Figure S6. ^{13}C NMR spectrum of 2-methyl-3-(2-nitro-vinyl)-1*H*-indole (**2**) in $CDCl_3$.

Table S3. NMR data of 9H-carbazole (**3**) in $CDCl_3$

Pos.	$\delta_{C(N)}$	XHn	δ_H	Mult.	COSY	H to C HMBC
1a, 8a	139.5	C				3, 6, 7, 2, 5, 4
3, 6	119.4	CH	7.25	m	7, 2, 8, 1, 5, 4	7, 2, 5, 4
4a, 5a	123.3	C				3, 6, 8, 1, 5, 4
5, 4	120.3	CH	8.10	m	3, 6	3, 6, 7, 2
2, 7	125.8	CH	7.43	m	3, 6, 8, 1	3, 6, 8, 1, 5, 4
1, 8	110.5	CH	7.44	m	9, 3, 6, 7, 2	3, 6, 5, 4
9	(108.5)	NH	8.11	br s	8, 1	

$^1H/^{13}C$ at 500.3/125.8 MHz

Figure S7. Correlations in the 2D NMR spectra of 9H-carbazole (**3**) in $CDCl_3$

(blue arrows = COSY, green arrows = HMBC).

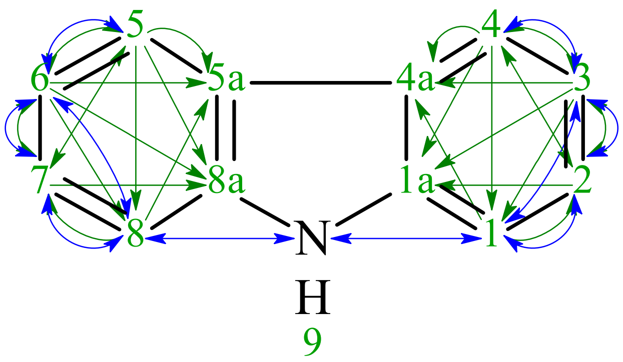

12/18/2017 12:50:46

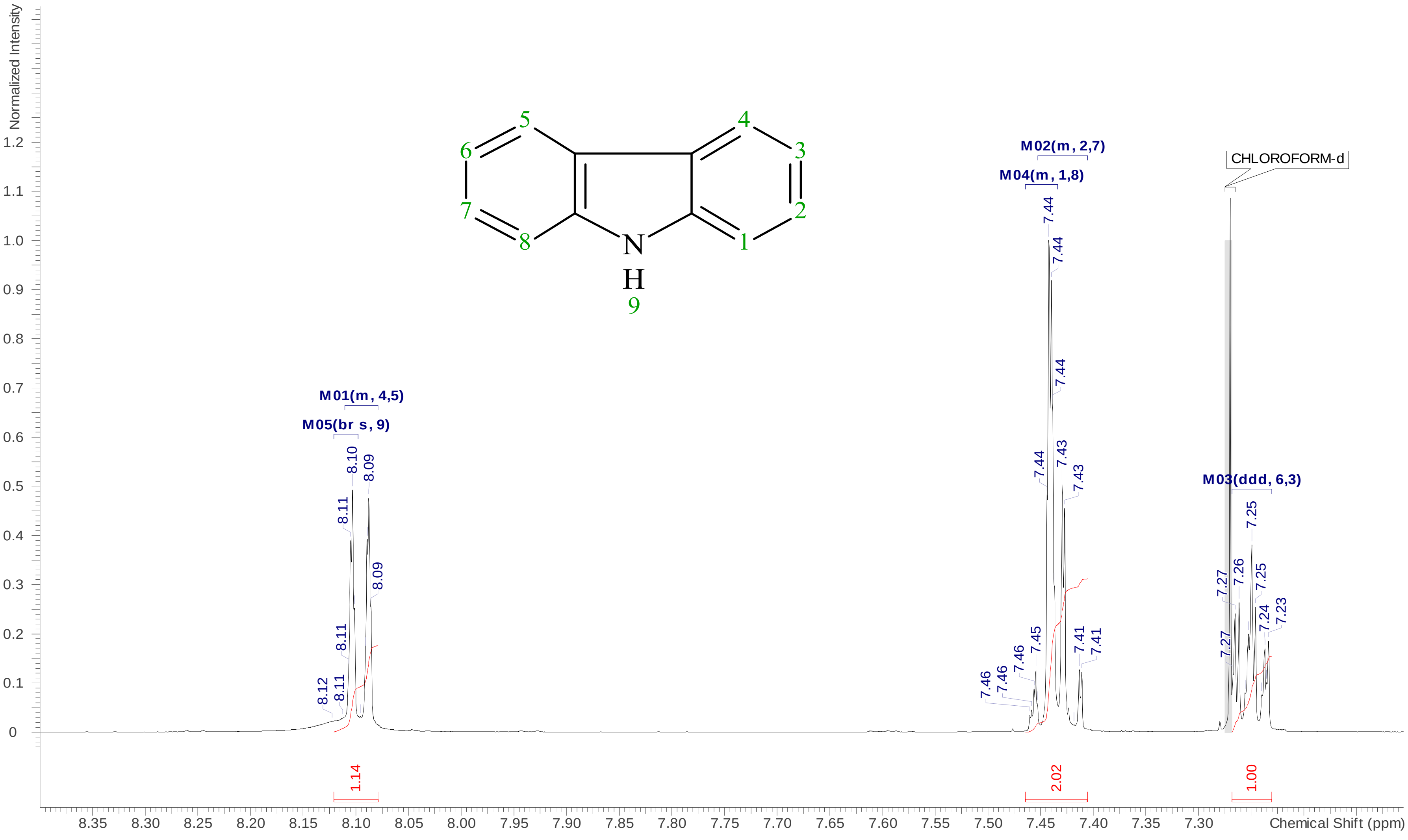

Figure S8. ^{1}H NMR spectrum of 9H-carbazole (**3**) in $CDCl_3$ (500.3 MHz)

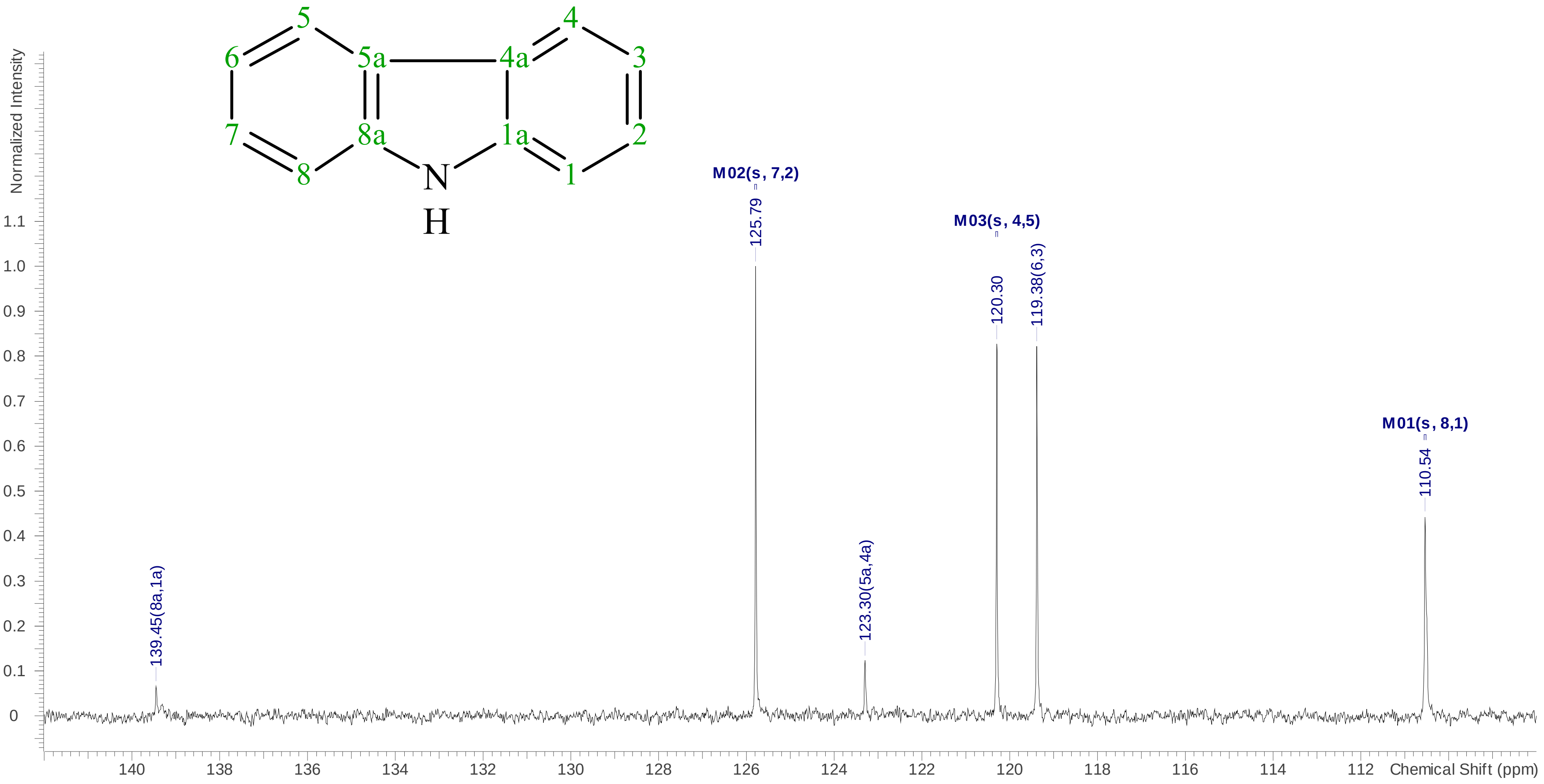

Figure S9. ^{13}C NMR spectrum of 9H-carbazole (**3**) in $CDCl_3$ (125.8 MHz)

Table S 4. NMR Data of 3-chloro-9*H*-carbazole (**4**) in CD_3OD ($^1H/^{13}C$ 500.3/125.8 MHz)

Atom#	C Shift	XHn	H Shift	H Multiplicity	COSY	H to C HMBC	C to H HMBC
1	109.0	NH				10, 6	
2	139.9	C				11, 13	
3	125.7	C				10, 9	
4	123.6	C				8, 6, 13	
5	142.3	C				7, 9	
6	112.1	CH	7.44	dt (8.1, 0.9)	7	8, 9	8, 4
7	127.5	CH	7.39	ddd (8.1, 7.0, 1.2)	8, 6	8, 9	9, 5
8	120.2	CH	7.16	ddd (7.9, 7.0, 1.1)	7, 9	6	6, 9, 4, 7
9	121.3	CH	8.03	dt (7.8, 0.8)	8	8, 7	6, 3, 7, 5
10	113.0	CH	7.40	dd (8.7, 0.5)	11		12, 3, 11
11	126.6	CH	7.32	dd (8.5, 2.1)	10, 13	10, 13	13, 12, 2
12	125.2	C				11, 10, 13	
13	120.7	CH	8.03	dd (2.1, 0.4)	11	11	4, 12, 11, 2

Figure S10. Correlations in the 2D NMR spectra of 3-chloro-9*H*-carbazole (**4**) in CD_3OD.

(blue and red COSY, green HMBC)

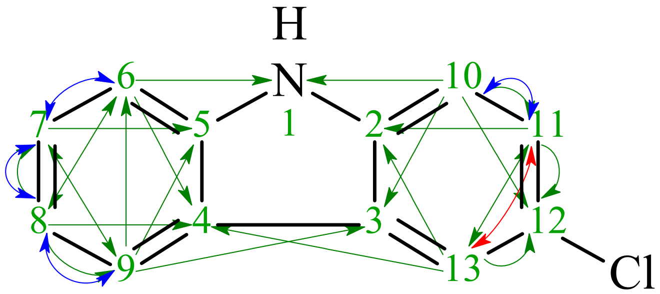

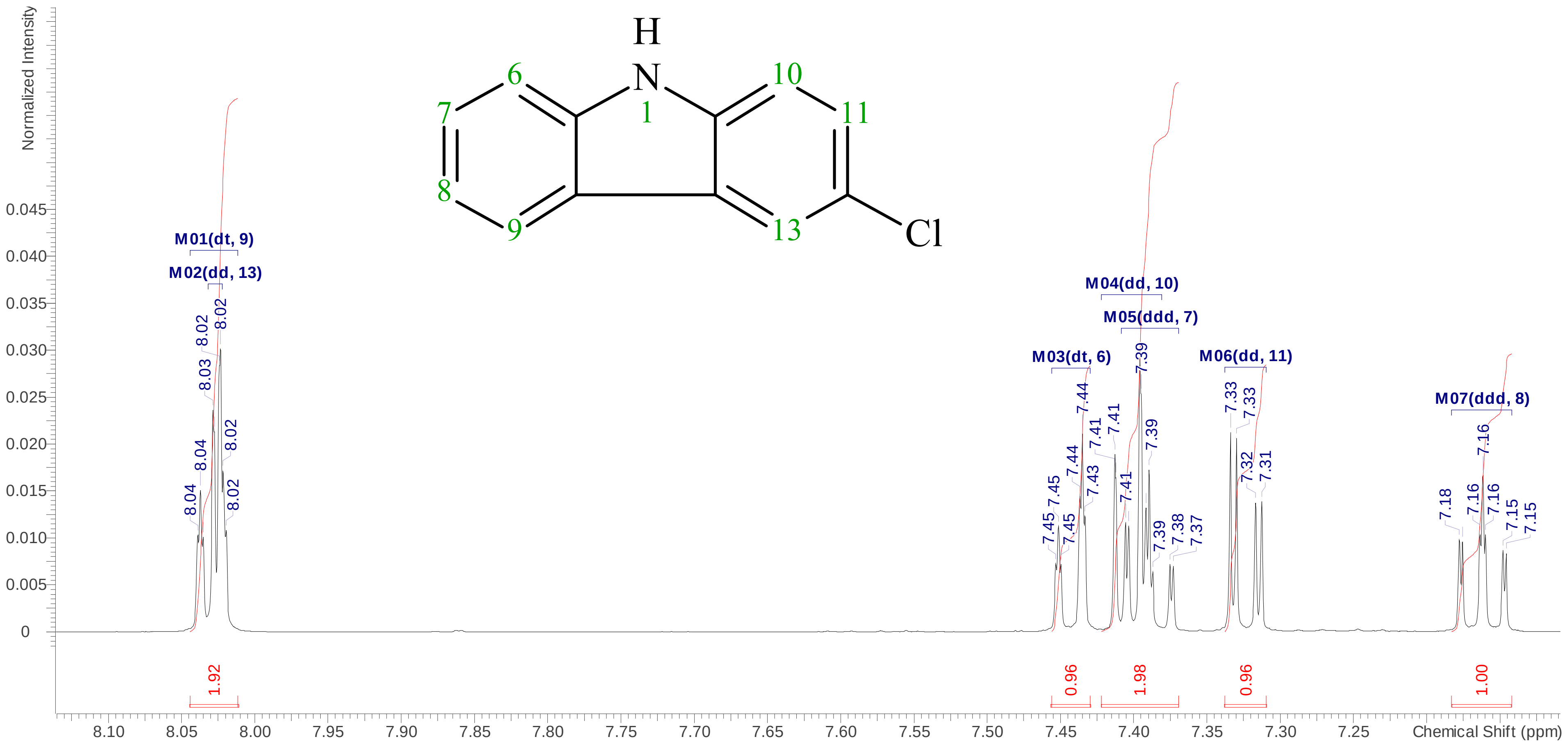

Figure S11. ^{1}H NMR spectrum of 3-chloro-9*H*-carbazole (**4**) in CD_3OD (500.3 MHz)

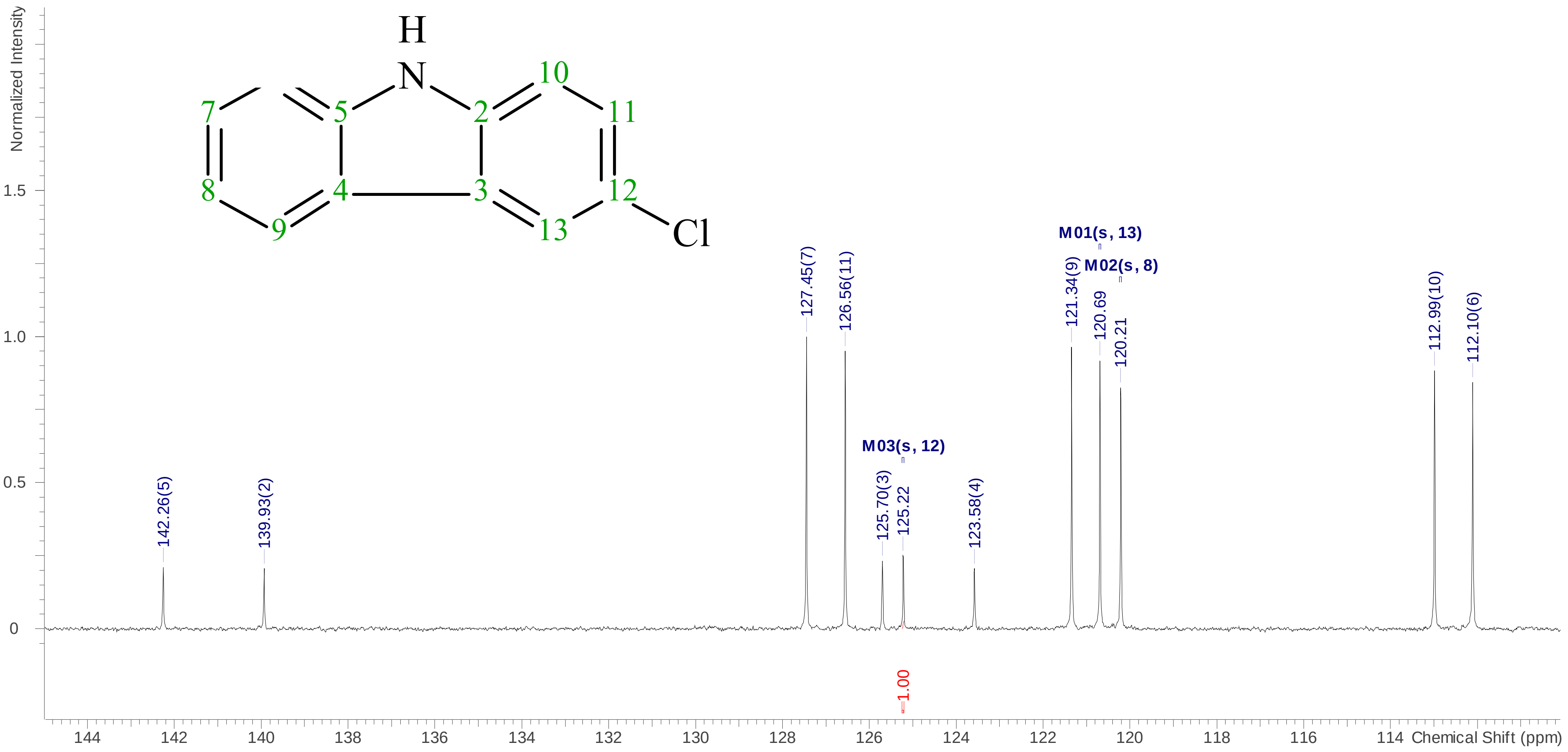

Figure S12. ^{13}C NMR spectrum of 3-chloro-9*H*-carbazole (**4**) in CD_3OD (125.8 MHz)

Table S 5. NMR Data of 4-hydroxymethyl-quinoline (**5**)

C Atom#	C Shift	XHn	H Shift	H Multiplicity	COSY	H to C HMBC	C to H HMBC
11	61.479	CH2	5.240	d (0.76)	3	3	3, 5, 4
3	118.130	CH	7.565	dd (5.42, 0.99)	11, 2	11, 2	11, 2
6	122.854	CH	7.961	dd (8.39, 0.76)	7.58	7.72	8, 10
5	125.767	C				11, 7, 9	
7	126.812	CH	7.577	td (7.02, 7.02, 1.22)	8, 6		5, 9
8	129.462	CH	7.723	ddd (8.39, 6.94, 1.30)	7, 9	6	6, 10
9	129.629	CH	8.142	d (8.54)	8	7	5
4	146.866	C				11	
10	147.313	C				8, 6, 2	
2	150.026	CH	8.850	d (4.43)	3	3	3, 10
1		N				11, 3, 9, 2	

Figure S13. Correlations in the 2D NMR spectra of 4-hydroxymethyl-quinoline (**5**) in CD_3OD. (blue and red COSY, green HMBC)

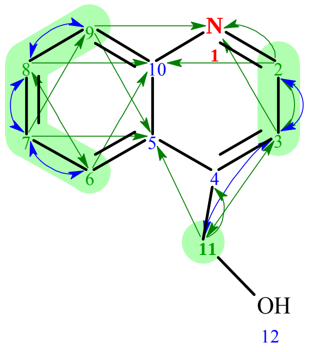

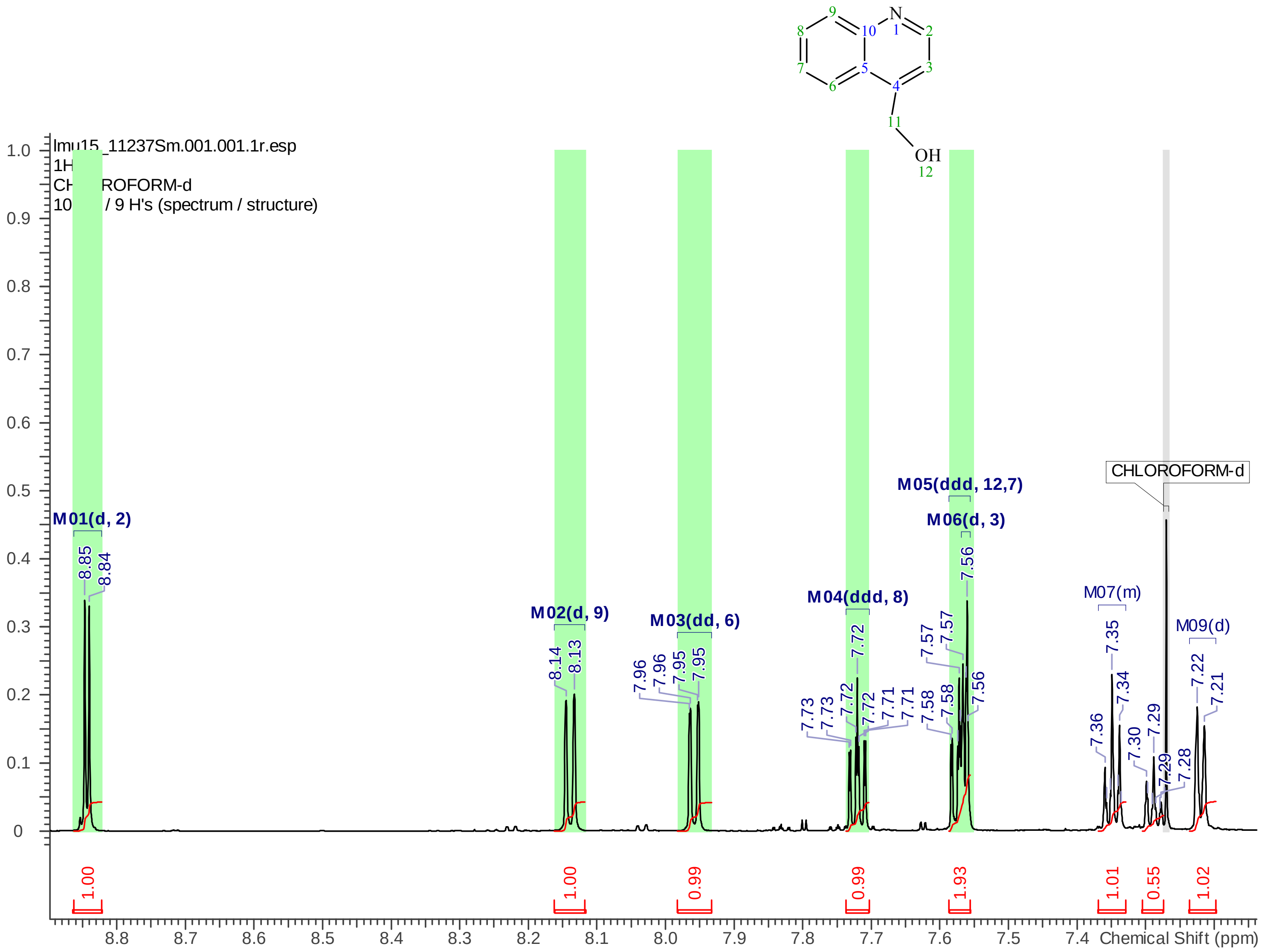

Figure S14. ^{1}H NMR spectrum of 4-hydroxymethyl-quinoline (**5**) in CD3OD (500.3 MHz)

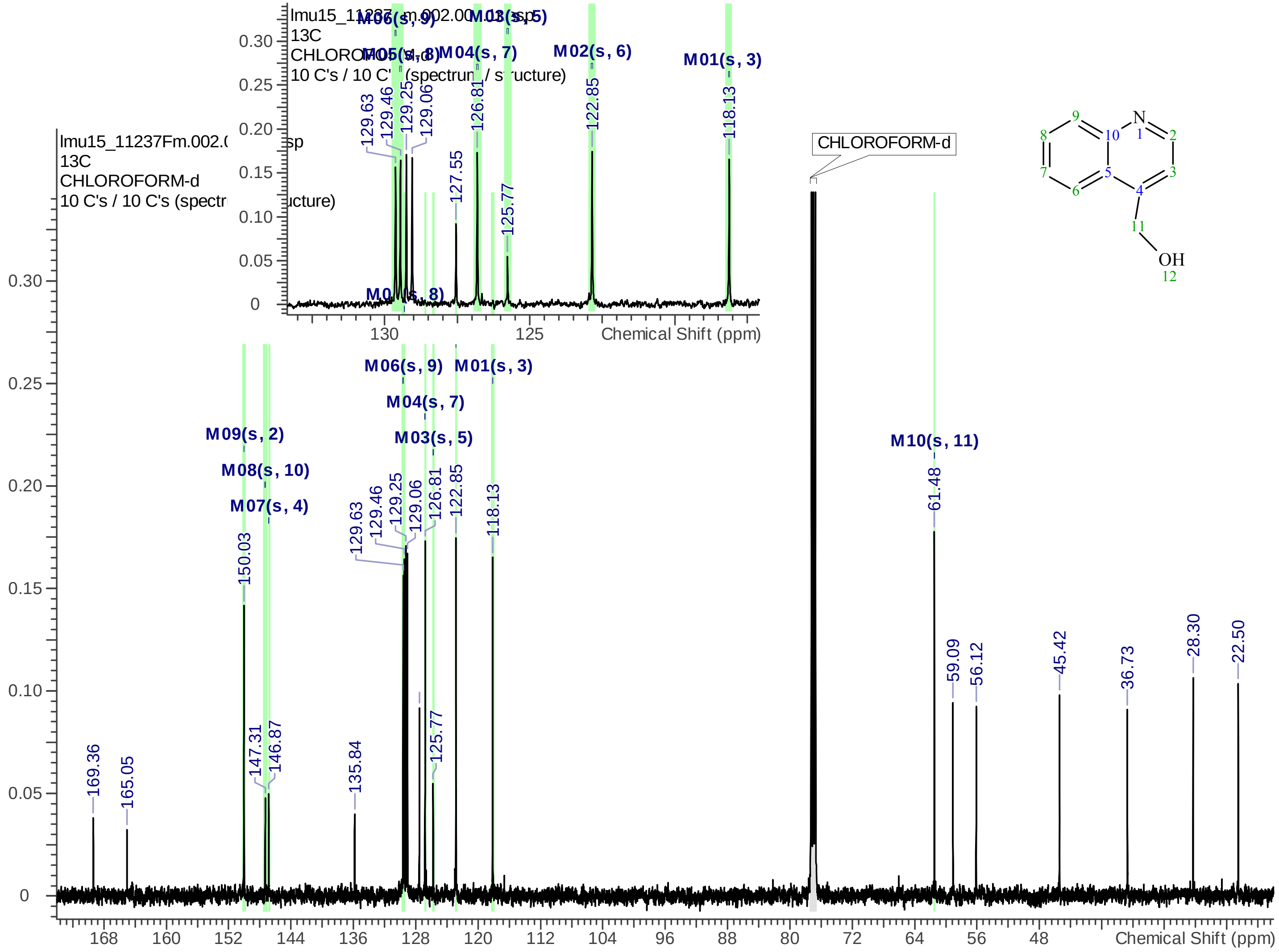

Figure S15. ^{13}C NMR spectrum of 4-hydroxymethyl-quinoline (**5**) in CD3OD. (500.3 MHz

Table S 6. NMR Data of 3,6-Dibenzylpyrazin-2(1*H*)-one (**6**) (^{1}H/^{13}C 500.3/125.8 MHz)

C Atom#	C Shift	XHn	H Shift	H Multiplicity	COSY	H to C HMBC	C to H HMBC
		NH	12.33	br s			
1	120.6	CH	7.10	m		3	
3	35.4	CH2	3.75	s			1, 9, 5, 4
3'	38.3	CH2	3.91	s	8', 6', 7'	8', 6', 7'	9', 5', 4', 1', 2'
1', 2'	156.0	C				3'	
4	137.4	C				9', 5', 3	
2, 4'	138.1	C				3', 8', 6', 7'	
7	126.1	CH	7.17	m	8, 6	8, 6	
8, 6	126.7	CH	7.23	m	7	9, 5, 7	7
9', 5'	128.5	CH	7.30	m		5,9	5,9
9, 5	128.7	CH	7.30	m		3	8, 6
8', 6', 7'	128.2	CH	7.24	m	3'		3', 4'

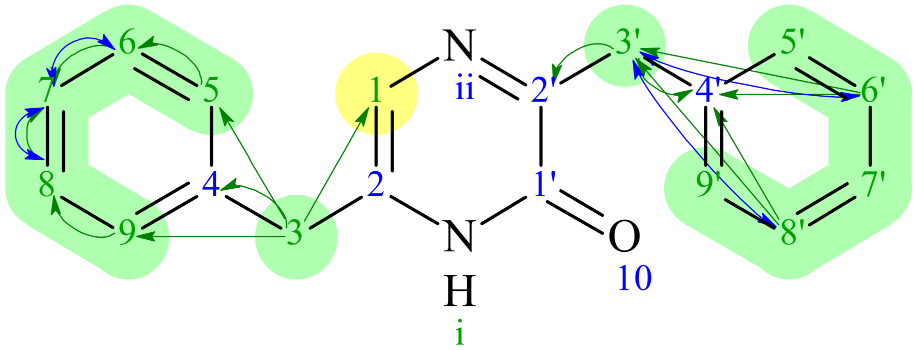

Figure S16. Correlations in the 2D NMR spectra of 3,6-Dibenzylpyrazin-2(1*H*)-one (**6**) in DMSO-d6.

(blue and red COSY, green HMBC)

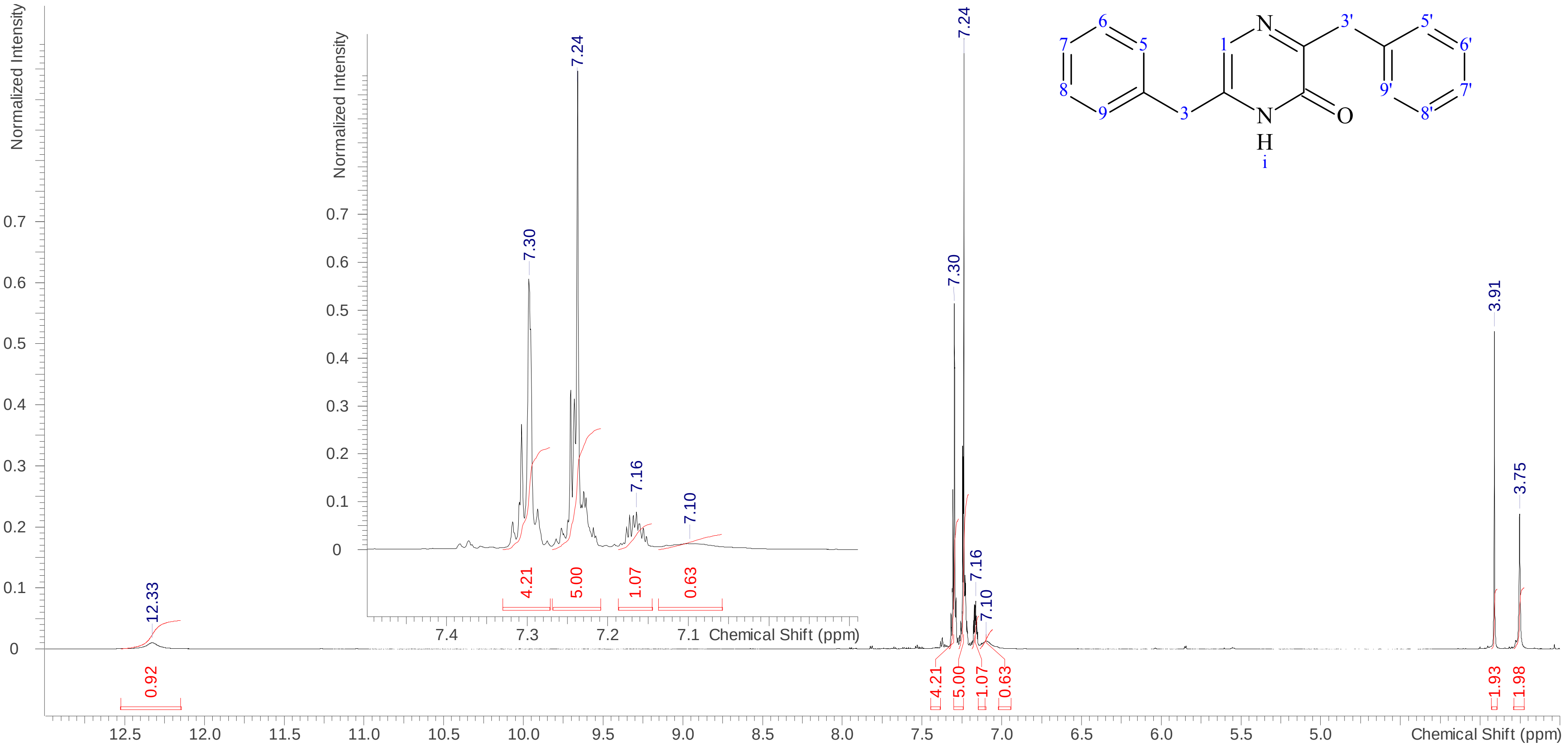

Figure S17. ^{1}H NMR spectrum of 3,6-dibenzylpyrazin-2(1H)-one (**6**) in DMSO-d_6 (700.4 MHz).

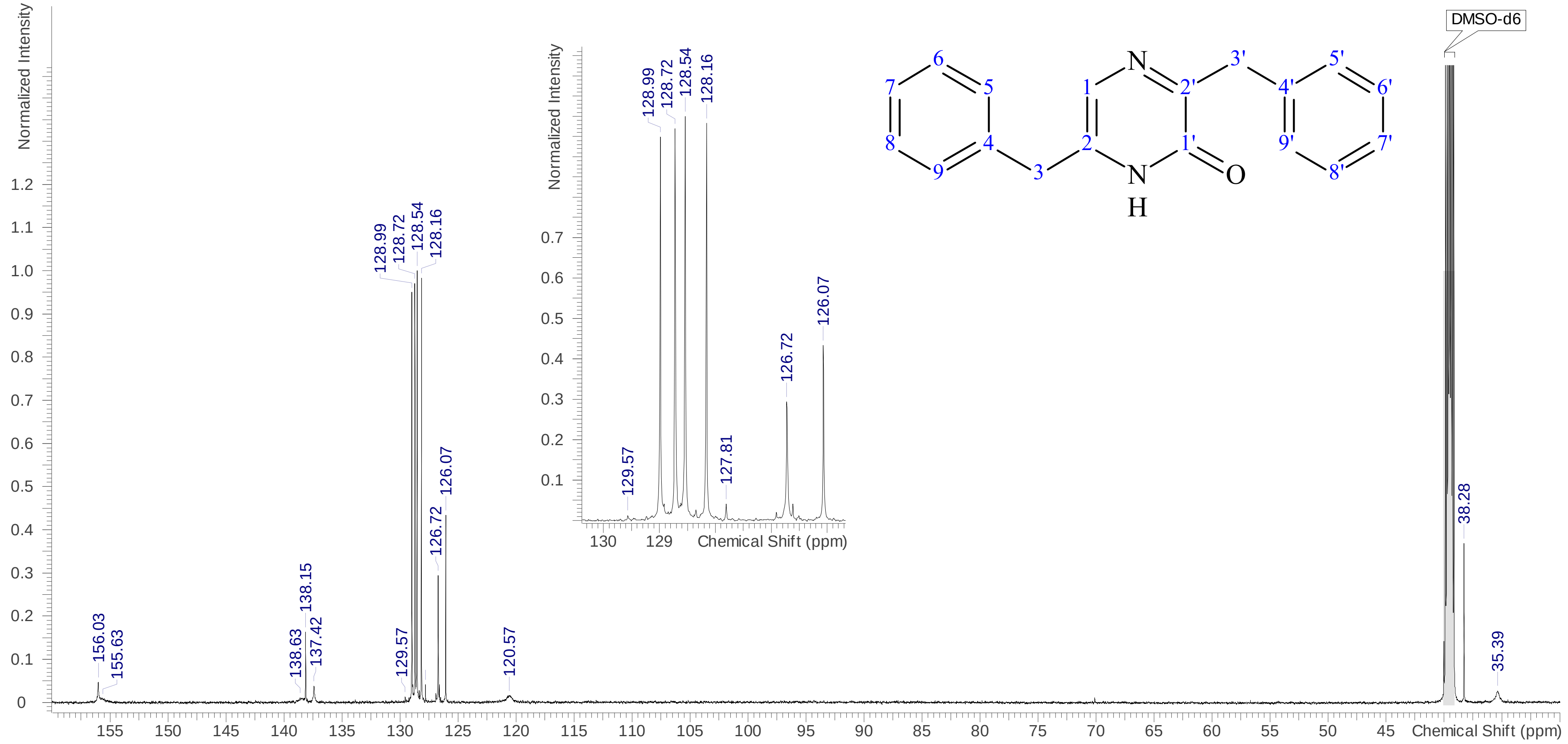

Figure S18. ^{13}C NMR spectrum of 3,6-dibenzylpyrazin-2(1H)-one (**6**) in DMSO-d_6 (176.1 MHz).

Figure S19. Antiviral activity against HCV (Hepatitis-C-Virus) Results

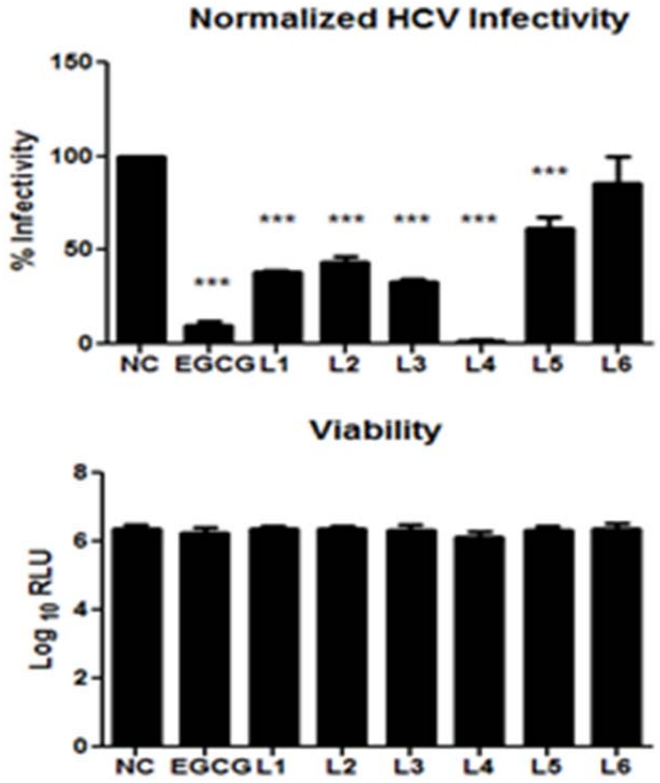

The assay was performed in quadruplicate (L1-L2) and triplicate (L3-L6) and is presented as the mean ± standard deviation. *** P ≤ 0.05.
